# Supplementary material for: Adsorption of Diclofenac Sodium by Aged Degradable and Non-Degradable Microplastics: Environmental Effects, Adsorption Mechanisms
Source: Toxics. 2022 Dec 27;11(1):24. doi: 10.3390/toxics11010024 (PMC9863539; doi:10.3390/toxics11010024)
Supplement: Supplementary file 1 [file toxics-11-00024-s001.zip › toxics-2103550-supplementary.pdf]

Supplementary Data

Adsorption of diclofenac sodium by aged degradable and non-degradable microplastics: Environmental effects, adsorption mechanisms

Siqi Liang [a], Kangkang Wang [a], Kefu Wang [a], Yuli Kou [a], Tao Wang [a], Changyan Guo [a,*], Wei Wang [b,*], Jide Wang [a,*]

Table S1. Structure and properties of the polymers of microplastics (MPs)

MPs	Molecule Structure	Polarity	PZC	m.p.
Polystyrene (PS)	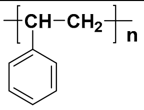	non-polar	4.86	166°C
Poly (butylene adipate-co-terephthalate) (PBAT)	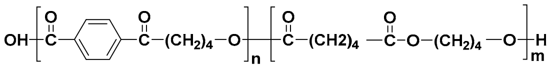	polar	3.92	130°C

Table S2. Structure and properties of the diclofenac (DCF)

Compound	Structure	log K_{ow}	pKa	Water Solubility	Molecular Weight
Diclofenac (DCF)	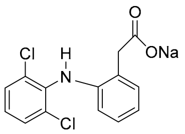	4.51	4.15	2.37 mg/L (25 ºC)	296.15 g/mol

Data obtained from Elizalde-Velazquez *et al.*, 2020.

Kinetic and adsorption isotherm models

At equilibrium, the mass q_e of contaminants adsorbed by MPs was calculated as follows:

$$q_e = \frac{(C_0 - C_e).V}{W}$$

where q_e is the adsorption capacity (mg/g), C_o and C_e are respectively the liquid phase concentrations of DCF in the initial and equilibrium states (mg/L), V (L) is the volume of the solution, and W (g/L) is the amount of MPs per liter of solution.

Both pseudo first-order kinetics models and pseudo secondary kinetics models are representative models that are now widely used to describe the kinetic processes of adsorption within solid-liquid systems. The linear form of pseudo first-order kinetics model is as follows:

$$\log(q_e - q_t) = \log q_e - \frac{K_1}{2.303} t$$

where K_1 (h^{-1}) is the rate constant of the pseudo first-order kinetics model.

The linear form of the pseudo-secondary model is as follows:

$$\frac{t}{q_e} = \frac{1}{K_2 {q_e}^2} + \frac{1}{q_e} t$$

where q_e (mg/g) is the the adsorption capacity at equilibrium and K_2 (g/mg·h) is the rate constants of the pseudo-secondary model.

Both the Langmuir model and the Freundlih model are representative models that are widely used to describe isothermal adsorption processes within solid-liquid systems. The linear equation of the Langmuir model can be expressed as follows:

$$q_e = \frac{q_{max} K C_e}{1 + K C_e}$$

where K (L/mg) is the Langmuir constant, q_{max}(mg/g) is the maximum adsorption capacity per unit surface of the adsorbent medium when saturation is reached.

The linear equation of the Freundlich model is as follows:

$$q_e = K_F {C_e}^n$$

where K_F (mg/g)(L/g)$^{1/n}$ stands for nonlinear adsorption capacity and n stands for nonlinear adsorption intensity.

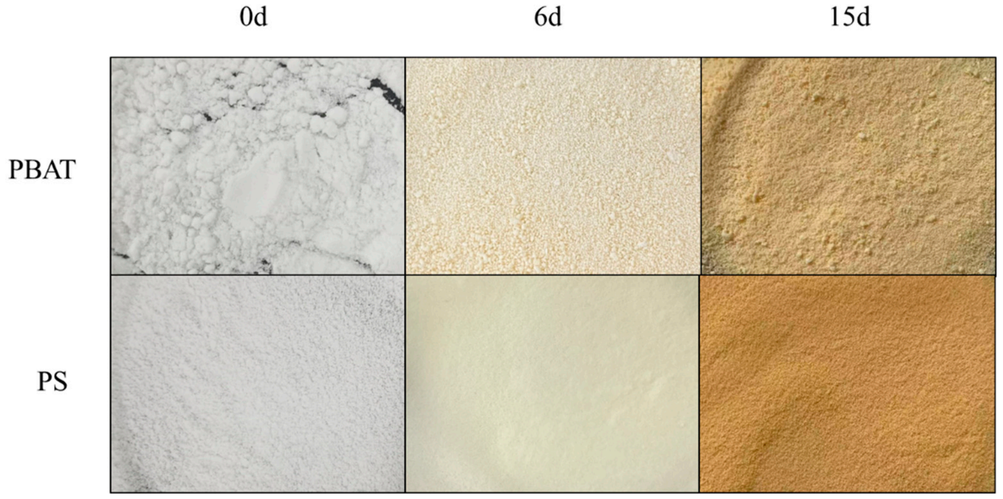

Figure S1. Macroscopic changes on the surface of microplastic particles before and after UV aging.

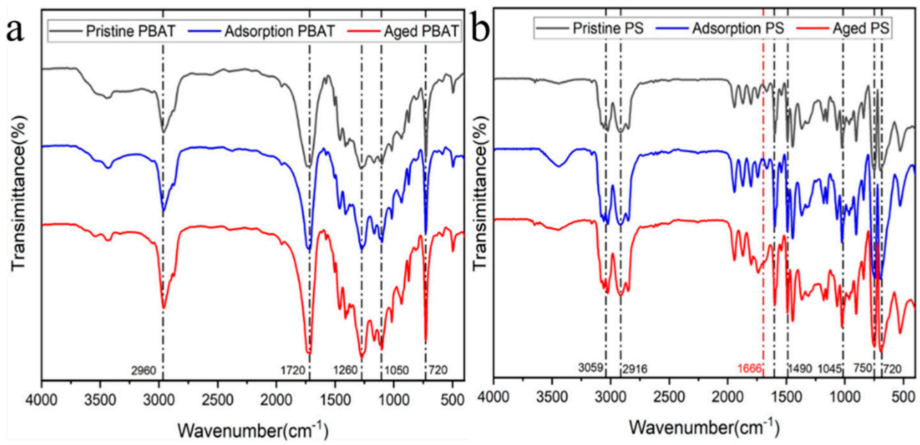

Figure S2. Fourier transform infrared spectra before and after UV aging and before and after adsorption（FTIR）（a. PBAT b. PS）

Table S3. Changes in oxygen content of PBAT and PS before and after UV aging.

Samples	Oxygen content (%)	
	PBAT	PS
Pristine	35.68±1.06	8.51±0.61
Aged	44.37±0.87	23.72±0.48

Table S4. Kinetic parameters of DCF adsorption by microplastics before and after aging

MPs	Pseudo-first order			Pseudo-second order		
	q_e(mg/g)	K_1(h^{-1})	R^2	q_e(mg/g)	K_2(g/(mg h))	R^2
PBAT	8.461±0.375	0.177±0.075	0.904	8.958±0.325	2.5222±0.665	0.958
PS	8.340±0.353	0.162±0.070	0.912	8.861±0.285	2.743±0.664	0.966

A-PBAT	26.843±1.563	0.718±0.056	0.924	29.655±1.550	0.496±0.115	0.966
A-PS	21.120±0.850	0.266±0.074	0.927	22.631±0.663	1.781±0.333	0.976

Table S5. Adsorption isotherm parameters of DCF on PBAT and PS before and after aging

MPs	Langmuir model			Freundlich model		
	q_e(mg/g)	K_L(L/mg)	R^2	n	K_F(mg/g)(L/g)$^{1/n}$	R^2
PBAT	24.518±1.606	2.604±0.678	0.983	25.756±1.879	2.853±0.495	0.990
PS	15.165±0.510	8.621±1.760	0.993	15.627±0.430	6.277±0.656	0.998
A-PBAT	31.016±0.665	4.192±0.535	0.984	31.591±0.343	4.299±0.343	0.996
A-PS	32.520±3.394	1.193±0.549	0.987	36.170±4.657	2.136±0.402	0.993

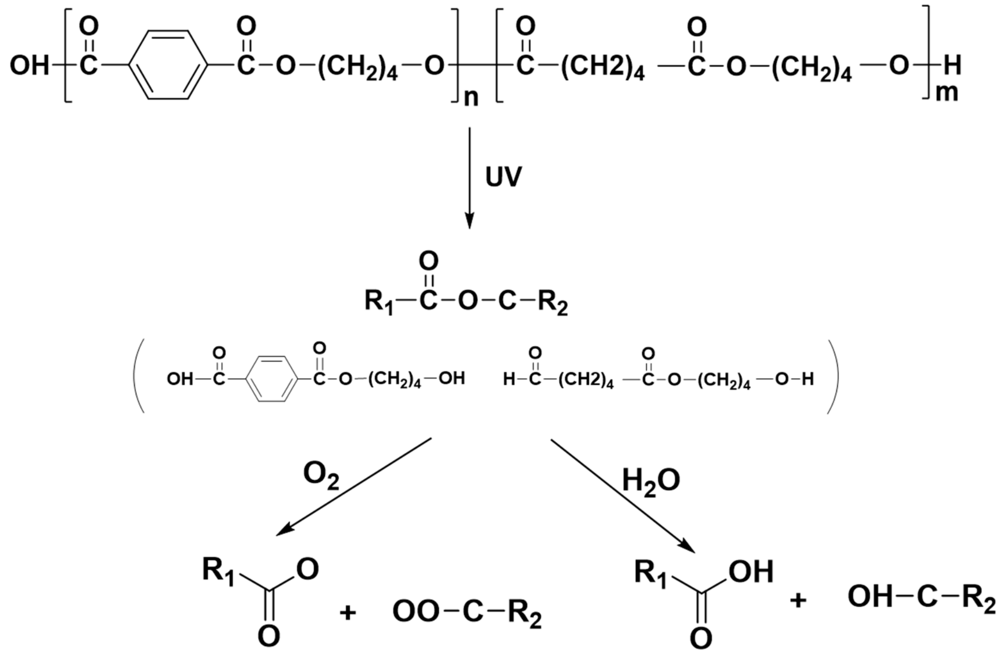

Figure S3. Aging process of PBAT

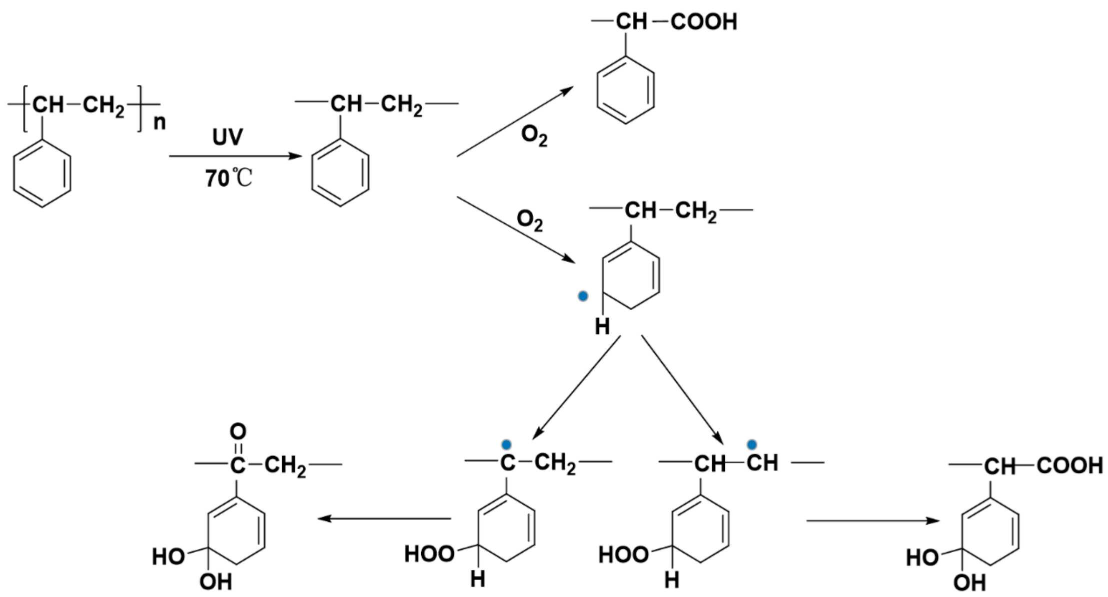

Figure S4. Aging process of PS
